# Characterization of Monte Carlo Dynamic/Kinetic Properties of Local Structure in Bond Fluctuation Model of Polymer System

**DOI:** 10.3390/ma14174962

**Published:** 2021-08-31

**Authors:** Wojciech Radosz, Grzegorz Pawlik, Antoni C. Mituś

**Affiliations:** Department of Theoretical Physics, Wroclaw University of Science and Technology, Wybrzeze Wyspianskiego 27, 50-370 Wroclaw, Poland; grzegorz.pawlik@pwr.edu.pl (G.P.); antoni.mitus@pwr.edu.pl (A.C.M.)

**Keywords:** local polymer structure, Monte Carlo bond fluctuation, time scales, inhomogeneous dynamics, complexity

## Abstract

We report the results of the characterization of local Monte Carlo (MC) dynamics of an equilibrium bond fluctuation model polymer matrix (BFM), in time interval typical for MC simulations of non-linear optical phenomena in host-guest systems. The study contributes to the physical picture of the dynamical aspects of *quasi-binary mosaic states* characterized previously in the static regime. The polymer dynamics was studied at three temperatures (below, above and close to the glass transition), using time-dependent generalization of the static parameters which characterize local free volume and local mobility of the matrix. Those parameters play the central role in the kinetic MC model of host-guest systems. The analysis was done in terms of the probability distributions of instantaneous and time-averaged local parameters. The main result is the characterization of time scales characteristic of various local structural processes. Slowing down effects close to the glass transition are clearly marked. The approach yields an elegant geometric criterion for the glass transition temperature. A simplified quantitative physical picture of the dynamics of guest molecules dispersed in BFM matrix at low temperatures offers a starting point for stochastic modeling of host-guest systems.

## 1. Introduction

### 1.1. Bond Fluctuation Model and Simulations of Physical Phenomena in Polymer Systems

Polymer systems are successfully used in various areas of science and technology. While a significant progress has been made in the theoretical statistical physics-based description of polymer physics [1], there still remain essential challenges related, in particular, to the characterization of static and dynamic features of local structure of the polymer matrix. Also, the origin of complex dynamics of physical phenomena in polymer-based systems close to the glass transition temperature remains unclear.

Computer simulations offer complementary theoretical tools for studies of polymer systems. Various simulation techniques are used, depending on the temporal and spatial scales relevant for the specific system [2,3,4].

In those cases when a large number of polymer chains is indispensable, Monte Carlo simulations use simplified models which neglect the detailed atomistic features of polymers. Amongst those models a special role is played by lattice bond fluctuation model (BFM) [5,6,7]. This non-specific model of polymer dynamics has given proof of its capability to predict/simulate a large variety of static and kinetic/dynamic effects in polymer materials like, e.g., properties of linear chains [8,9,10,11,12,13,14,15,16,17,18,19] and polymer rings [20,21], polymer blends and interfaces [22,23], gels and networks [24], localization of glass transition [25,26,27], polymer blends [28], (co)polymers at surfaces [29], polymer brushes in good solvents [30,31,32,33,34,35,36], polymer thin films [37,38,39], equilibrium polymers [17,40,41], general self-assembly [42,43,44], networks and gel point [45,46,47,48], olympic gels [49,50,51], hyperbranched polymers [52,53], dendrimers [54,55,56,57], lipid membranes [58,59,60,61], see also review papers [62,63,64]. Studies of dynamics of BFM chains included, among others, such topics as reptation predictions and scaling of relaxation times [7], Rouse and reptation dynamics at finite temperatures [65], crossover from Rouse to Reptation Dynamics [66], dynamical properties of the slithering-snake algorithm [67], or diffusion of single long polymers [68].

The generalized BFM model, which takes into account the interaction of external electromagnetic fields with guest molecules in a polymer matrix (host-guest system), has offered a theoretical description of a multitude of nonlinear optical phenomena, summarized in a review paper of this Special Issue [69]. This model is based on two concepts: local free volume in polymer matrix, quantified in terms of time-dependent local void parameter $V(\vec{r},t)$, and local mobility of the matrix, characterized by time-dependent local mobility parameter $C(\vec{r},t)$, see Section 2.2 and Section 2.3 for details. Those parameters are parts of the expressions for transition rates of kinetic/dynamic processes in host-guest systems like, e.g., photoisomerization and reorientation of guest molecules, promoted by light-matter interactions and thermal fluctuations [69], and, correspondingly, play an important role in Monte Carlo simulations of BFM host-guest systems. A detailed probabilistic characterization of random fields $V(\vec{r},t)$ and $C(\vec{r},t)$ in terms of adequate probability distributions and correlation functions constitutes one of challenges in the physics of model polymer systems.

### 1.2. Local Free Volume—Static Characterization of Mosaic-Like States

**Figure 1:**
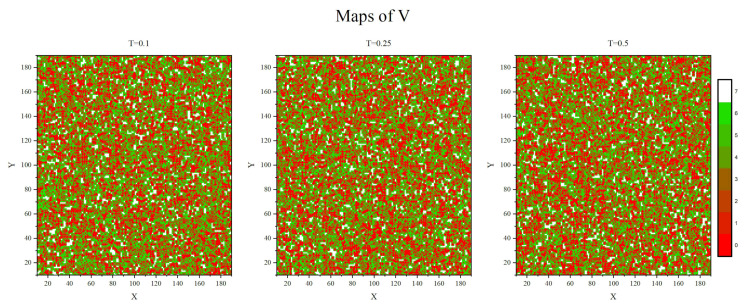
Instantaneous 2-dimensional maps of parameter *V*: T=0.1 (**left**), T=0.25 (close to glass transition, (**middle**)) and T=0.5 (**right**).

Figure 1 shows two-dimensional (2D) maps of parameter $V(\vec{r},t)$— instantaneous sections of 3D system—for three temperatures: below, close to and above glass transition temperature $T_{g}$. A typical map is a complex intertwining *mosaic* (originally proposed a long time ago [70]) of local areas (cells) occupied (red) and unoccupied (green) by monomers (see Section 2.2 for technical details).

The characterization of a typical instantaneous configuration of local free volume $V(\vec{r})$ in terms of *static* probability distributions and correlation functions, carried out on large, intermediate and local spatial scales, results in a simplified physical picture of *quasi-binary mosaic-like states* [69,71]. Approximately 50% of the cells are blocked in the following sense: there is a non-negligible amount of monomers in their closest neighbourhood. On the other hand, about one third cells are free or nearly free of monomers in their close neighbourhood. The latter are not distributed randomly in the space but display a tendency to group into scale-free (fractal) clusters with fractal dimension dependent on the size of a cluster.

An eye-inspection of the three configurations in Figure 1 does not reveal any differences between them—actually, all statistical distributions and correlation functions are practically independent on the temperature. Thus, the structure of *mosaic-like* states depends, first of all, on the density of the polymer matrix.

We point out that the *mosaic-like* spatial organization seems to be an inherent feature of dense condensed matter systems, which display some kind of local order in the absence of long-range correlations [72,73].

### 1.3. Heterogeneous Dynamics and Complexity

The dynamics of guest molecules immersed in the matrix or attached to the polymer chains, depends, among others, on steric hindrances imposed by the polymer matrix, characterized by field $V(\vec{r})$. The cells marked red in Figure 1 are blocked and the dynamics of guest molecules which occupy those cells is strongly hindered. This kind of restricted dynamics is referred to as a *trapping* event. On the contrary, the dynamics of guest molecules in cells marked green depends solely on their interactions with external fields. The resulting dynamics becomes heterogeneous in space, reflecting the heterogeneity of the distribution of local free volume.

The dynamics of the polymer matrix results in the time-dependence of the local free volume: $V=V(\vec{r},t)$. It modifies the dynamics of trapping events which become random variables characterized by the spectrum of trapping-time periods. Correspondingly, the dynamics of guest molecules becomes heterogeneous both in space and time. This effect can promote complex behaviour of the host-guest system. Namely, theoretical studies, not related to the polymer physics, lead to the general conclusion that the long-tailed distribution (power law) of trapping-time periods promotes stretched exponential complexity [74,75,76,77,78]. This scenario was demonstrated for the 2D Lennard-Jones liquid close to the melting temperature: the long-tailed distribution of the intervals of trapping events has promoted the stretched exponential behaviour of correlation functions which characterize topological features of *mosaic-like states* [72,73].

Complex dynamics was reported in recent BFM modeling of all-optical poling [79], Surface Relief Grating inscription [80,81], and dynamics of model polymer chains functionalized with azo-dye molecules in two [82] and three [80] dimensions. It was tentatively ascribed to the physical picture presented above, in which the broad spectrum of temporal scales characterizing the times of life of clusters of blocked and free cells determines an overall dynamics of host-guest systems.

### 1.4. Objective

The qualitative physical picture of a heterogeneous spatio-temporal dynamics presented above needs quantitative confirmation. So far, only the static spatial heterogeneity was analyzed and, at least partially, confirmed [69,71].

The goal of the paper is to make the first steps towards a detailed probabilistic characterization of the dynamics of local free volume, hypothetically responsible for complex dynamics of physical phenomena in model polymer-based systems. To this end we develop methods and tools which generalize their counterparts used for the characterization of static configurations. Particular emphasis is placed on characterization of the field $V(\vec{r},t)$; other fields, in particular the local mobility field $C(\vec{r},t)$, are analyzed to a lesser extent.

The paper is organized as follows. The next Section summarizes briefly the main features of bond fluctuation Monte Carlo model and introduces static and dynamic parameters which characterize locally model polymer matrix. Third Section is devoted to the probabilistic analysis of chosen aspects of Monte Carlo dynamics of local structure of BFM matrix. In the last Section we discuss briefly an emerging physical picture of local dynamics in the polymer matrix.

## 2. Materials and Methods

The methods and concepts used for the simulations and for an analysis of polymer’s local structure were worked out in detail in the review paper of this Special Issue [69]; in what follows we discuss only their milestones.

### 2.1. Monte Carlo Bond Fluctuation Model

Bond fluctuation model of a polymer chain stems from the bead-spring model [1]. The chain is composed of effective units called monomers located on a simple cubic lattice, connected by bonds with variable lengths equal (in lattice constants) $2,\sqrt{5},\sqrt{6},3,3,$$\mathrm{and}\sqrt{10}$ [5,9,64]. The corresponding potential energies read $E=E_{0}$ for the three shortest bonds and E=0 for the longer ones. Parameter $E_{0}$ sets the energy scale and defines the reduced temperature $T^{*}=k_{B}TE_{0}$, where $k_{B}$ and *T* denote, respectively, Boltzmann constant and an absolute temperature. In what follows $T^{*}$ is referred to as *T*.

**Figure 2:**
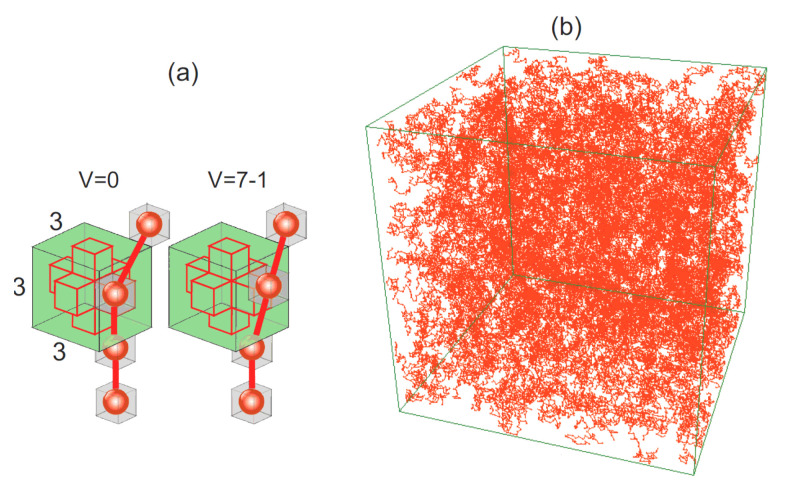
(**a**) Parameter *V*—a scheme of calculations. (**b**) Snapshot of the simulation: configuration of polymer system. 10% of all polymer chains are shown.

The polymer matrix consisted of N=24,000 polymer chains, each with M=20 monomers, see Figure 2b. The size of the simulation box read (in lattice constants) $V_{p}=200^{3}$ [2,9,71]; the reduced density $\rho =8\;N\;M/V_{p}=0.48$ characterizes a concentrated polymer solution [9]. Periodic boundary conditions were used.

The polymer matrix was simulated using standard Metropolis Monte Carlo method [2,83]. In a single Monte Carlo Step (MCS) each of the monomers performed a trial move of a unit length along randomly chosen direction x,y or *z*. The move was accepted under the following conditions [5,69]: (i) the lengths of emerging bonds belonged to the fixed set of lengths, (ii) the move did not violate any steric limitations and (iii) the move was accepted by Metropolis acceptance rule [83]. The configurations of the polymer matrix were prepared in the same way as in Ref. [71]. After the equilibration period the data for further analysis were sampled in the time interval $\Delta t=2\cdot 10^{5}$ MCS. This specific choice of the simulation interval was dictated by the fact that it is sufficient to observe chosen nonlinear optical phenomena in BFM host-guest systems like, e.g., surface relief gratings formation [80,82] ($\Delta t\approx 4\cdot 10^{4}$ MCS), all-optical poling [79] ($\Delta t=2\cdot 10^{5}$ MCS) and others [71,84,85,86,87,88]. It is important to stress that a different choice of the time of the simulation would influence the results. The simulations were done for three representative temperatures T=0.1,0.25and0.5 corresponding, respectively, to glassy phase, close neighbourhood of glass transition and liquid phase. The glass transition temperature was estimated as $T_{g}\approx 0.23-0.26$ [71,79].

MC method, basically applied for the simulations of equilibrium systems, can be also used to study the dynamics or non-equilibrium systems. Namely, the MC-time evolution of coarse-grained systems does not contradict real dynamics under the condition that the trial moves are limited to local conformational changes [2,89]. Thus, we interpret the results of MC simulations in terms of dynamical parameters.

### 2.2. Local Void Parameter $V(\vec{r},t)$

The local void parameter $V(\vec{r,t})$ characterizes the degree of local inhomogeneity of the distribution of monomers around lattice point $\vec{r}$ at time *t*. The quantification of this concept is based on the occupancy of a 3×3×3 cube with center at lattice point $\vec{r}$, see Figure 2a. The central cross consists of the cell at lattice point $\vec{r}$ and its 6 nearest neighbours. If a monomer occupies a position inside the cross then the central cell is considered as blocked and the value of local void parameter is set to zero: $V(\vec{r,t})=0$. Otherwise $V\left(\vec{r},t\right)=V_{max}-k$, where $0\leq k\leq V_{max}$ is the number of monomers inside the cube. Thus, parameter *V* characterizes the strength of steric hindrances—they become important for low values of *V* and, on the other hand, negligible for its high values. We have used $V_{max}=7$, which is the maximal occupancy of a 3×3×3 cube by monomers found in the simulations [69,71,90]. The cube used in the modeling corresponds approximately to the volume $10\;{\mathrm{nm}}^{3}$ for PMMA chains [69,71].

### 2.3. Local Mobility Parameter $C(\vec{r},t)$

Local mobility parameter $C(\vec{r},t)$ characterizes the local dynamics of a polymer matrix around lattice point $\vec{r}$ at time *t* in terms of an evolution of the occupancies of lattice sites by monomers inside the cube with center at $\vec{r}$, in a single MC step: [69,85,90]

(1) $$C\left(\vec{r},t\right)=\sum_{i}\left|n_{i}\left(t+1\right)-n_{i}\left(t\right)\right|,$$

where $n_{i}\left(t\right)$ denotes the occupancy of *i*-th cell at time *t*: $n_{i}=1$ when it is occupied by a monomer, otherwise $n_{i}=0$. The sum extends over cells in a 9×9×9 cube—the large size of the cube is dictated by the fact that monomers in the system are relatively sparse.

### 2.4. Parameter $D(\vec{r},t)$

Finally, we introduce the parameter

(2) $$D\left(\vec{r},t\right)=V\left(\vec{r},t\right)\cdot C\left(\vec{r},t\right)$$

which combines two features of a polymer matrix: free volume and the dynamics around lattice site $\vec{r}$. It characterizes the transition rate for rotational diffusion of guest molecules driven by thermal mobility of the polymer chains [69,79,85,90,91].

## 3. Results

### 3.1. Evolution in Time of Local Free Volume: Parameter $V(\vec{r},t)$

The macroscopic dynamics of guest molecules is determined by two main factors related to the polymer matrix: the distribution of local free volume in space and its temporal evolution. The latter is characterized by the times of life $t_{L}\left(V,\vec{r}\right)$, i.e., the intervals of time in which parameter *V* remains constant for the cell at $\vec{r}$. In particular, $t_{L}\left(V=6\right)$ characterizes the periods of local free dynamics and $t_{L}\left(V=0\right)$—the periods of trapping events.

**Figure 3:**
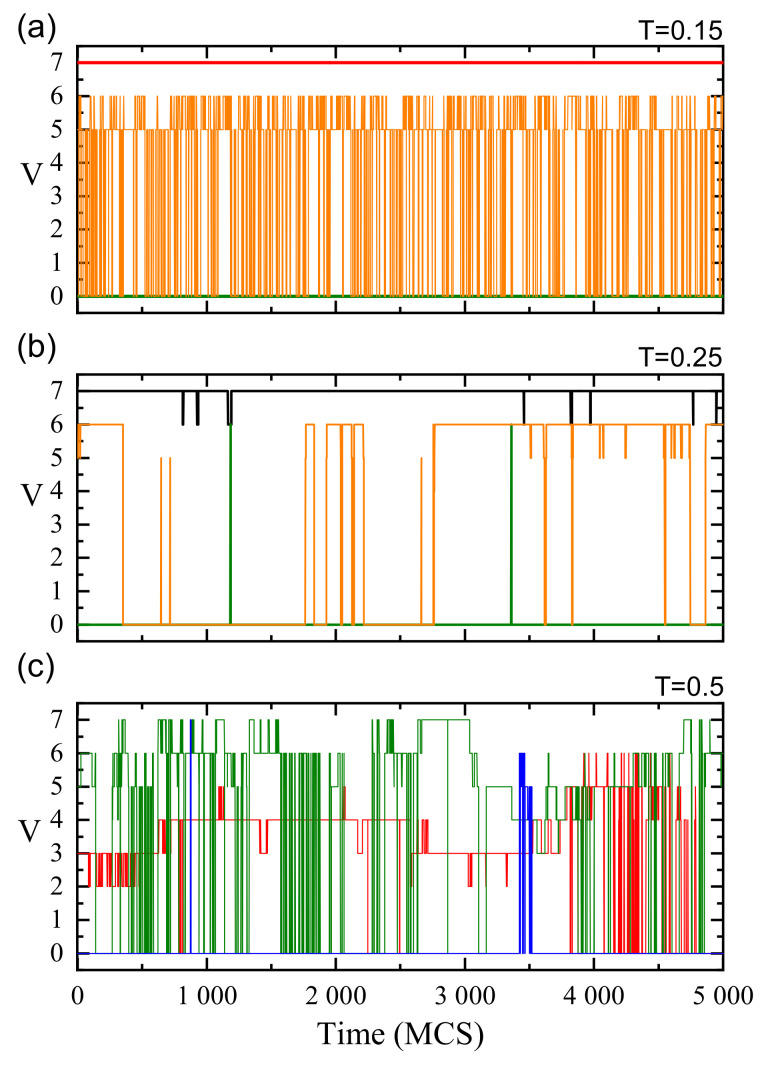
Time-dependence of local free volume *V* for three representative cells at T=0.15 (**a**), 0.25 (**b**) and 0.5 (**c**), see text for more details.

The dynamics of local free volume displays a variety of patterns depending on the temperature. Figure 3 shows typical trajectories V(t) for MC-time interval of $5\times 10^{3}$ MCS. In spite of the fact that it constitutes only a few percent of the typical time interval in which non-linear optical effects were observed in the simulations [69], it is sufficient for the demonstration of various types of local dynamics of the matrix.

For T=0.15, well below $T_{g}$, most of local free volume is frozen (panel (a), V=7 and V=0, red and green lines, respectively). A low concentration of cells display rapid transitions between locally blocked (V=0) and (nearly) free regimes (V=5,6). So, there are two typical patterns of local dynamics, corresponding to long (dominating) and short temporal scales (times of life).

Close to the glass transition temperature the dynamics displays a large degree of diversity (panel (b)). Long-lived local free voids (V=6,7—black line) are present, as well as long-lived blocked cells (V=0, green line) which change their state twice, for a very short period, to a nearly free one (V=6,7). A very different pattern of the dynamics corresponds to the oscillations between blocked and (nearly) free states (red line). In this case the times of life do not display any typical (regular) pattern. Other kinds of the dynamics (not shown) were also found. We conclude, rather tentatively, that a wide spectrum of temporal scales (times of life), from the shortest to the longest ones, is present.

At higher temperatures (T=0.5) the dynamical patterns which were found both at low temperature and close to the glass transition are present: oscillations between blocked and free states with very short as well as with long times of life and, on the other hand, long-lasting trapping events with rapid changes of parameter *V*. We believe that a wide spectrum of times of life is also present in this case. On the other hand, both cases are *qualitatively* different. This topic is briefly discussed in Section 4.

To quantify the aforementioned qualitative conclusions two approaches were used. The first one concerns the characterization of times of life $t_{L}\left(V\right)$ of local voids with fixed value of parameter *V*. To this end, the times of life of such cells were monitored during the whole simulation ($\Delta t=2\cdot 10^{5}$ MCS) and the empirical histograms, representing the probability distribution $\rho (t_{L},V)$, were constructed. This kind of analysis provides an information about the *maximal* as well as *typical* time scales related to local dynamics of free volume.

**Figure 4:**
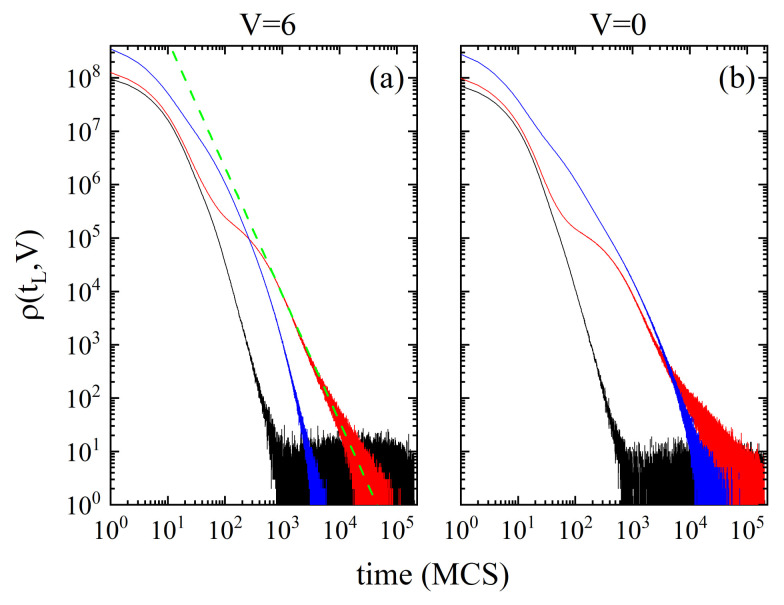
Double logarithmic plot of non-normalized probability distribution $\rho (t_{L},V)$ for V=6 (**a**) and V=0 (**b**). T=0.1 (black line), T=0.25 (red line) and T=0.5 (blue line). Green dashed line represents power law.

Figure 4 shows the plots of $\rho (t_{L},V)$ against MC time for V=0 and V=6. This choice is dictated by their exceptional properties [71,79]—the corresponding cells form the two main components of *quasi-binary mosaic-like states*. Consider first the case V=6 (Figure 4a). Such cells are nearly free of monomers in their closest neighbourhood and group into scale-free (fractal) clusters [69,71]. At low temperature (T=0.1) a smooth decrease of the probability distribution is followed, starting from $t_{L}\approx 10^{3}$ MCS, by an irregular behaviour corresponding to longer times of life, up to $2\cdot 10^{5}$ MCS (i.e., the time of the simulation). We point out that the plot is incomplete—its missing part corresponds to a large concentration (around 44%) of V=6 cells with $t_{L}>2\cdot 10^{5}$ MCS. This situation is typical for MC simulations at low temperatures, and is caused by weak thermal fluctuations of the polymer matrix. For a high temperature (T=0.5) a smooth decay of the probability distribution is present, with a maximal value of $t_{L}$ around $4\cdot 10^{3}$ MCS. Close to $T_{g}$ (T=0.25) much longer times of life are present, up to $t_{L}\approx 10^{5}$ MCS. The plot displays a new feature: it has two inflection points which mark two different regimes. In the first one the plot matches the plot for T=0.1. In the second regime some features characteristic for a power law (over 2 but not 3 decades) are present:

(3) $$\rho \left(t_{L},T\approx T_{g}\right)∼t_{L}^{\alpha },$$

with α≈−2.38. The origin of the power law can be ascribed to the slowing down mechanism.

The probability distributions $\rho (t_{L},0)$ for the cells with V=0, which constitute the blocked part of the matrix in the *quasi-binary mosaic-like states*, bear a strong resemblance to their counterparts for V=6, with one significant exception: the cells with V=0 display longer temporal scales than the cells with V=6. Namely, the times of life $t_{L}$ reach the values up to $6\cdot 10^{4}$ MCS for T=0.5 and up to $2\cdot 10^{5}$ MCS close to the glass transition temperature. As was the case with V=6, at low temperature a vast majority (around 78%) of V=0 cells are of long-lived ($t_{L}>2\cdot 10^{5}$ MCS).

**Figure 5:**
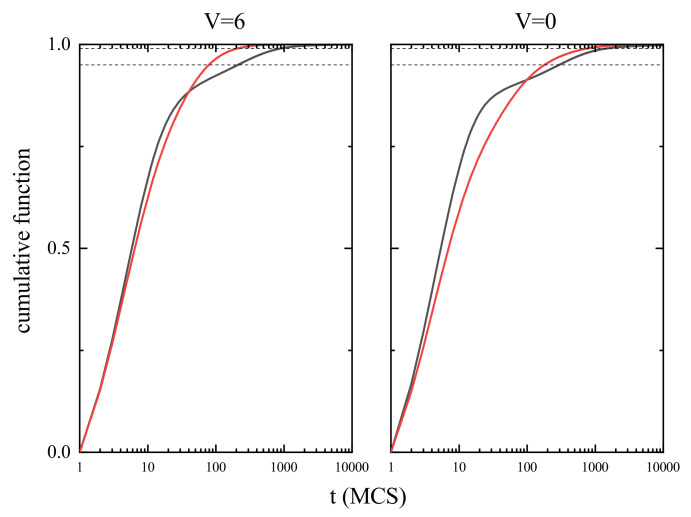
Empirical cumulative functions F(V,t) for V=6 (**left**) and V=0 (**right**). T=0.25 (blue line) and T=0.5 (red line). Horizontal lines mark the values 0.95 and 0.99.

The *typical* time scales correspond to the times of life $t_{L}$ of the *majority* of the cells. They can be inferred from the cumulative distribution function

(4) $$F\left(V,t\right)=\int_{0}^{t}\rho \left(V,t_{L}\right)dt_{L},$$

shown in Figure 5 for V=6 and V=0. In both cases the time of life for the majority of the cells does not exceeds $10^{3}$ MCS. For example, at high temperature (T=0.5) 95% of the cells with V=6 have $t_{L}<70$ MCS and for 99% of the cells $t_{L}<200$ MCS. For V=0 one observes a slight increase of the corresponding times of life—200 MCS and 300 MCS, respectively. Close to the glass transition temperature the times of life are approximately three times larger.

The second method of the characterization of local dynamics of parameter *V* concerns its evolution in a fixed cell $\vec{r}$, in the full simulation period. To this end we define the time average of parameter $V(\vec{r},t)$:

(5) $$\bar{V\left(\vec{r}\right)}=\frac{1}{\Delta t}\sum_{k=1}^{\Delta t}V\left(\vec{r},t_{k}\right).$$

**Figure 6:**
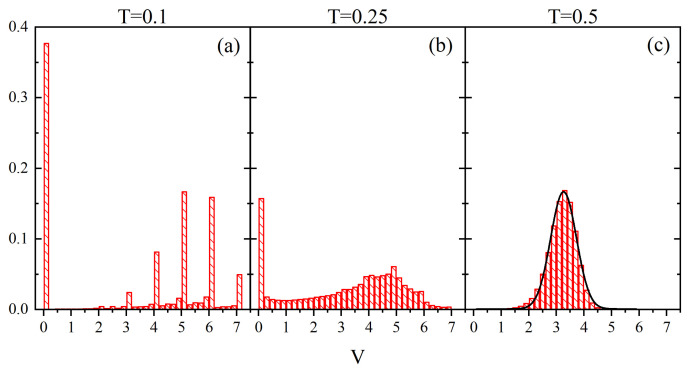
Normalized probability density $\rho (\bar{V\left(\vec{r}\right)})$ for T=0.1 (**a**), T=0.25 (**b**) and T=0.5 (**c**). Solid line (T=0.5) represents gaussian fit.

Figure 6 shows normalized empirical histograms which represent the probability density $\rho (\bar{V\left(\vec{r}\right)})$, calculated from all the cells in the system. Clearly, three distinct regimes are present. At low temperatures (T=0.1, Figure 6a), the distribution displays peaks for integer values of *V*, with negligibly small bars otherwise. The heights of the peaks reproduce the probability distribution $\rho_{S}\left(V\right)$ of random variable in a static configuration *V* [69,71]. Correspondingly, the vast majority of the cells are frozen in the time interval Δt, reflecting, in particular, the presence of a very long tail of times of life for V=6 discussed above. Close to the glass transition (T=0.25, Figure 6b) a new feature appears: the probability distribution becomes smooth, with the exception of the peak at V=0. The remaining part displays a flat maximum in the interval V=4−5. Finally, in the high temperature regime (T=0.5, Figure 6c) the probability density becomes nearly gaussian, with a maximum at V≈3.2 and standard deviation σ≈0.47.

The latter result offers a semi-quantitative estimation based on the central limit theorem [92], of a typical period of time in which the parameter *V* remains constant in a cell at high temperature (T=0.5). Namely, if the realizations of a random variable *V* in subsequent MCS were independent, the probability density $\rho (\bar{V\left(\vec{r}\right)})$ would be gaussian with the standard deviation $\sigma_{S}/\sqrt{\Delta t}\approx 0.01$, where $\sigma_{S}=2.7$ denotes the standard deviation for the static probability distribution $\rho_{S}\left(V\right)$. The actual value σ≈0.47 is much larger, as an effect of the correlations in time between the values of *V* at a given lattice point. Let us assume, for simplicity, that *V* remains constant in time interval τ, and that the random variables *V* in two such intervals are statistically independent. This simple (rather naive) model yields the following estimation of parameter τ. The number $N_{G}$ of independent random variables which, when added together, yield the gaussian probability density, satisfies the equation $\sigma =\sigma_{S}/\sqrt{N_{G}}$. One finds $N_{G}\approx 34$, which yields an estimation of the block length in which *V* remains constant: $\tau =\Delta t/N_{G}\approx 7500$ MCS.

A short discussion of the time scales reported above and their relation to the characteristic times in which non-linear effects are observed in the MC simulations of host-guest systems can be found in Section 4.

### 3.2. Evolution in Time of Local Mobility Parameter $C(\vec{r},t)$

**Figure 7:**
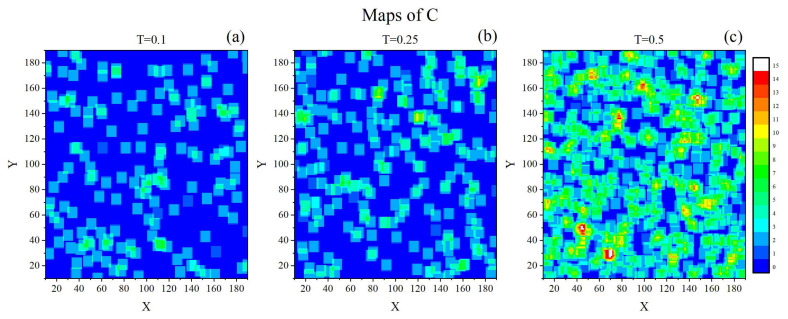
Instantaneous 2-dimensional maps of parameter *C*: T=0.1 (**a**), T=0.25 (**b**) and T=0.5 (**c**).

Figure 7 shows exemplary instantaneous 2-dimensional maps of parameter *C* for three temperatures: T=0.1,0.25 and 0.5. At low temperature the vast majority of the cells, referred to as *immobile*, are not influenced by the thermal motions of the chains. The *mobile* cells, i.e., those with a higher mobility of the chains in their neighbourhood, are rather sporadic and scattered throughout the system. Close to the glass transition the map matches the low-temperature map with the only difference that the concentration of mobile cells is slightly higher and some traces of spatial correlations between them are present. At high temperature mobile cells dominate the system and form a complex spatially correlated structure.

**Figure 8:**
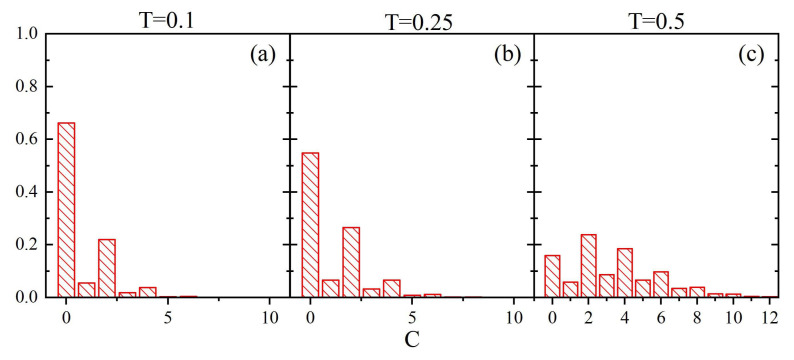
Empirical probability distributions ρ(C): T=0.1 (**a**), T=0.25 (**b**) and T=0.5 (**c**).

Those observations are fully supported by empirical probability distributions ρ(C) calculated from a single realization of the configuration $C(\vec{r},t)$, shown in Figure 8. At low and intermediate temperatures, the dominant mobility corresponds to C=2, while at a higher temperature a wider spectrum of values *C* (up to C=15) is present.

The local dynamics of parameter *C* in a fixed cell $\vec{r}$, in the full simulation period, was studied in the same way as for parameter *V*. Namely, we define the time average of parameter $C(\vec{r},t)$:

(6) $$\bar{C\left(\vec{r}\right)}=\frac{1}{\Delta t}\sum_{k=1}^{\Delta t}C\left(\vec{r},t_{k}\right).$$

**Figure 9:**
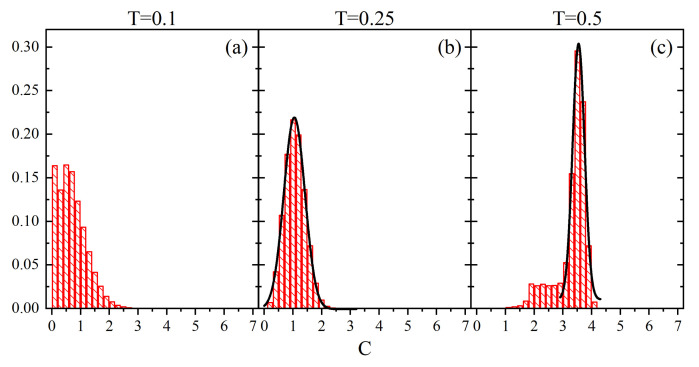
Normalized probability density $\rho (\bar{C\left(\vec{r}\right)})$ for T=0.1 (**a**), T=0.25 (**b**) and T=0.5 (**c**). Solid lines represent gaussian fits (see text).

Figure 9 shows the normalized empirical probability distributions of $\bar{C\left(\vec{r}\right)}$. Unlike the case with parameter *V*, the low-temperature probability distribution is rather smooth and does not reproduce the probability mass function ρ(C). The difference between the two cases stems from different sizes of cubes used in the definitions for *V* and *C*. Namely, since the latter is much larger, a change of parameter *C* is not obligatorily accompanied by the change of local free volume *V*. Close to the glass transition temperature an interesting effect appears—the probability distribution clearly differs from the low-temperature distribution, in spite of the fact that the corresponding probability distributions ρ(C) are very similar (Figure 8). It reflects the presence of different time scales in both cases.

A semi-quantitative estimation of characteristic time scales was done using the simple approach based on central limit theorem, introduced in previous Section. Close to the glass transition the probability distribution is reasonably well fitted using gaussian function (solid line in Figure 9, middle panel). The number of independent random variables reads $N_{G}\approx 15$, which yields an estimation of the block length in which *C* remains constant: $\tau =\Delta t/N_{G}\approx 1.7\cdot 10^{4}$ MCS. At high temperature the probability distribution displays a sharp peak accompanied by a low-probability shoulder. The gaussian fitting procedure of the peak (solid line in Figure 9, right panel) yields $N_{G}\approx 140$ and τ≈1800 MCS. It is worth making two comments. Firstly, the characteristic time scale at T=0.5 is of an order of magnitude shorter than close (T=0.25) to the glass transition. Two phenomena bring this effect on: a higher mobility at higher temperature and critical slowing down at glass transition, the latter observed in MC simulations of an all-optical poling [79]. Secondly, the time scale at T=0.5 is of the same order of magnitude (actually it is four times shorter) than the time scale characteristic for parameter *V* at the same temperature.

### 3.3. Parameter $D(\vec{r},t)$

Parameter $D(\vec{r},t)$, which characterizes a cumulative effect of local free volume and local mobility on the orientational diffusion of guest molecules at point $\vec{r}$ at time *t*, inherits main features of its both components. Low values indicate that the diffusion at point $\vec{r}$ is hindered, because of a small amount of local free volume or low level of local mobility. On the contrary, strong diffusion is promoted by high values of both parameters. A moderate local diffusion corresponds to the cases when one of the parameters has a high value and the second—a low value, see below for more details.

**Figure 10:**
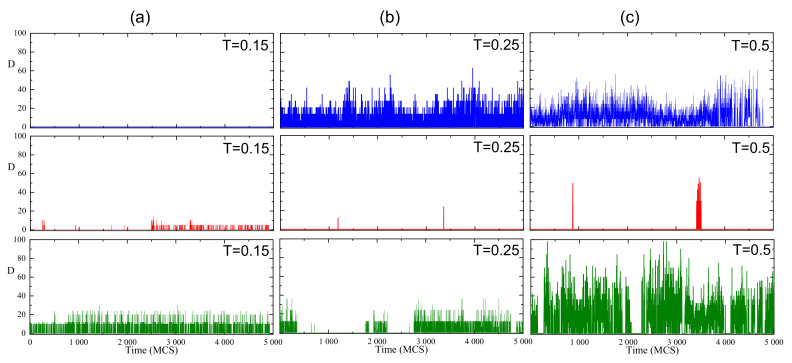
Time-dependence of parameter *D* in three representative cells for T=0.15 (**a**), 0.25 (**b**) and 0.5 (**c**).

Figure 10 shows the evolution in time of parameter $D(\vec{r},t)$ for three representative cells, at three temperatures. In what follows we interpret the results in terms of a rotational diffusion promoted by the temporal evolution of this field. At a low temperature the maximal value of *D* corresponds to V=7 and C≈5, thus $D_{max}\approx 35$. The first cell represents the case without an orientational diffusion. In the second cell a weak diffusion is present in a half of the short simulation period, with D≈5. Third cell displays a moderate diffusion interrupted by short periods of a high activity, with D≈20. Close to the glass transition parameter $D_{max}$ reads approximately 7·6=42 (see Figure 8). A large variety of patterns of temporal evolution are present, including lack of diffusion, moderate/strong diffusion in the simulation period, as well as periods of moderate diffusion separated by periods of strongly hindered diffusion. The case of a high temperature ($D_{max}\approx 100$) is similar, with one exception—no alternating periods of activity/inactivity were observed.

**Figure 11:**
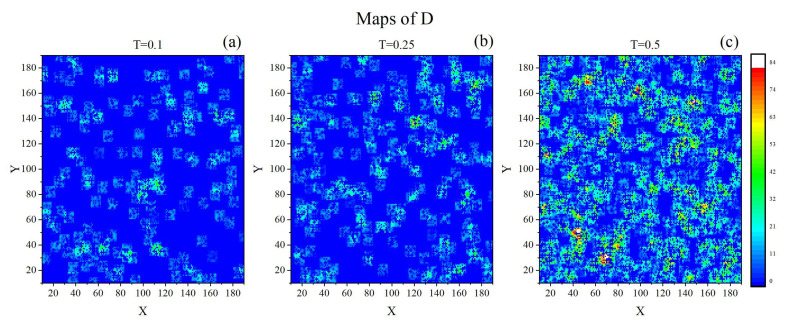
Instantaneous 2-dimensional maps of parameter *D*: T=0.1 (**a**), T=0.25 (**b**) and T=0.5 (**c**).

**Figure 12:**
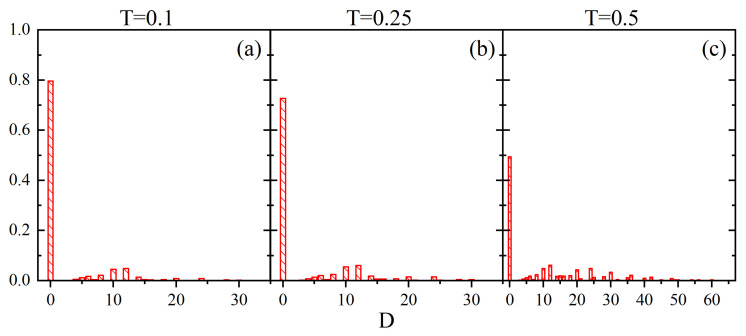
Empirical probability distributions ρ(D): T=0.1 (**a**), T=0.25 (**b**) and T=0.5 (**c**).

The field $D(\vec{r},t)$ is strongly inhomogeneous in space, see Figure 11, which shows its instantaneous 2D sections/maps. As expected, we find a striking qualitative similarity with the maps of the field $C(\vec{r},t)$ shown in Figure 7. The inhomogeneity is characterized, as in the case of parameter *C*, in terms of the normalized empirical probability distributions ρ(D), shown in Figure 12. They are dominated by the peaks at D=0, which originate from the peaks at V=0 [69,71] and C=0 (Figure 8). At low temperature and close to the glass transition around 75−80% of the cells do not support orientational diffusion. At high temperature the D=0 peak is mainly due to the V=0 peak which corresponds to approximately 40% of blocked cells. The probabilities of non-zero values of *D* are small; the probability distribution displays some features of a uniform distribution in the interval $0<D<D_{max}$.

The local dynamics of parameter *D* in a fixed cell $\vec{r}$, in the full simulation period, was studied using the time average of parameter $D(\vec{r},t)$:

(7) $$\bar{D\left(\vec{r}\right)}=\frac{1}{\Delta t}\sum_{k=1}^{\Delta t}D\left(\vec{r},t_{k}\right).$$

**Figure 13:**
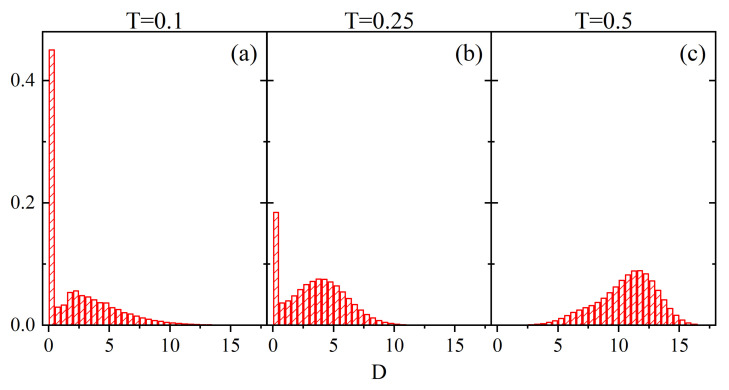
Normalized empirical probability distributions $\rho (\bar{D\left(\vec{r}\right)})$ for T=0.1 (**a**), T=0.25 (**b**) and T=0.5 (**c**).

Figure 13 shows the normalized empirical probability distributions $\rho (\bar{D\left(\vec{r}\right)})$. As expected, they inherit some characteristic features of histograms from Figure 6 and Figure 9: a sharp peak at $\bar{D\left(\vec{r}\right)}=0$ and an asymmetry of the plots. The former plays an important role in the localization of the glass transition temperature discussed in the next Section.

### 3.4. Localization of Glass Transition

The MC dynamics of local free volume marks a narrow interval of temperatures around glass transition temperature. Two approaches were used. In the first one we study a global parameter *D* being an average 〈...〉 over the whole system of local parameter $\bar{D\left(\vec{r}\right)}$:

(8) $$D=\langle \bar{D\left(\vec{r}\right)}\rangle =\frac{1}{L^{3}}\sum_{i=1}^{L^{3}}\left(\bar{D\left(\vec{r_{i}}\right)}\right).$$

**Figure 14:**
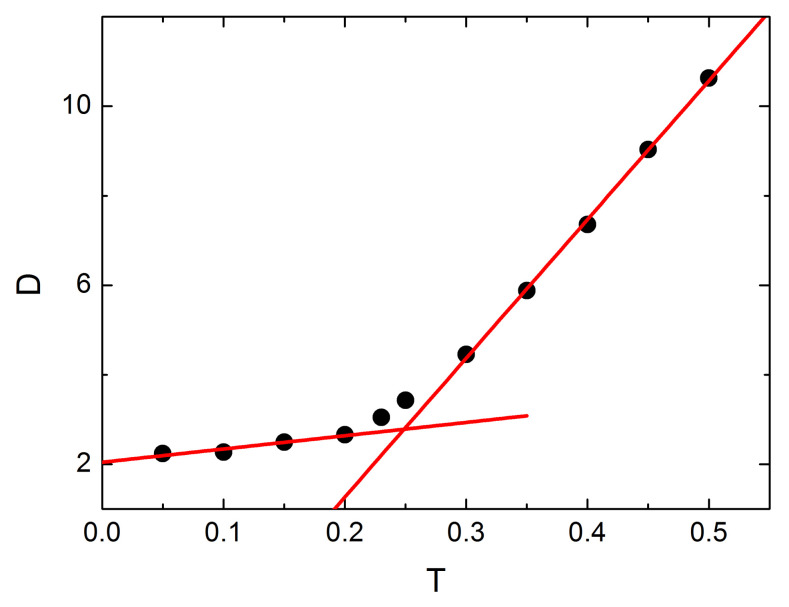
Temperature dependence of parameter *D*, Equation (8). Solid lines represent linear fits.

Figure 14 shows the plot of temperature dependence of parameter *D*. Two linear regimes, for low and high temperatures, are separated by a non-linear regime in the interval T=0.2−0.3. The linear fits intersect at T=0.25, clearly marking the change of the regimes, and providing an estimation of $T_{g}$. It is in a good agreement with previous estimations of $T_{g}$ based on MC simulations of geometric parameters like square radius of gyration ($T_{g}=0.26$) or mean free volume ($T_{g}=0.25$), physical parameters ($T_{g}=0.23$ for mean bond energy) and non-linear optical processes (angular hole burning yields $T_{g}=0.25-0.29$, all-optical poling—$T_{g}=0.22-0.25$) [71,79].

**Figure 15:**
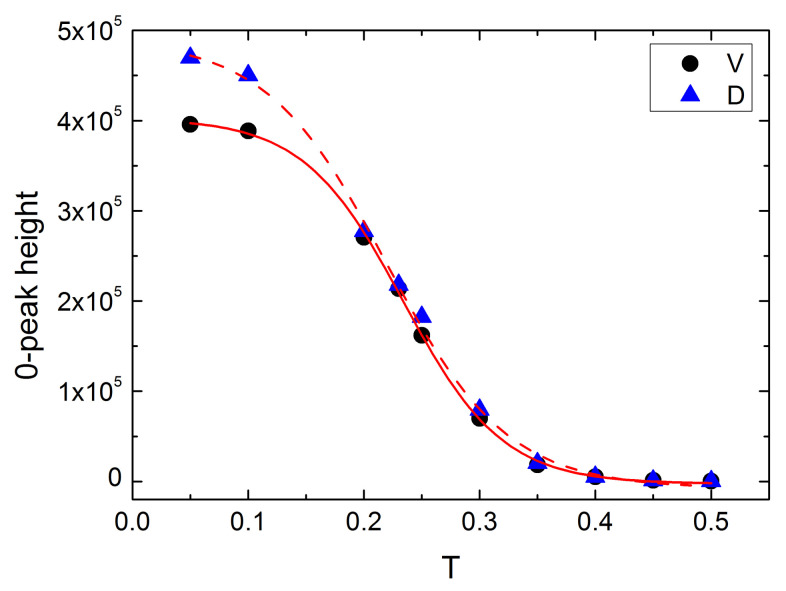
Plots of the amplitudes of the peaks for $\bar{V\left(\vec{r}\right)}=0$ (black circles) and $\bar{D\left(\vec{r}\right)}=0$ (blue triangles) against temperature. Red solid and dashed lines represent Boltzmann fits, Equation (9).

The second approach, based on the probability densities $\rho (\bar{V\left(\vec{r}\right)})$ (Figure 6) and $\rho (\bar{D\left(\vec{r}\right)})$ (Figure 13), offers an elegant, geometric-based criterion for the glass transition temperature. Namely, both plots display sharp peaks located at $\bar{V\left(\vec{r}\right)}=\bar{D\left(\vec{r}\right)}=0$, which corresponds to the *blocked* (V=0) part of the matrix. The amplitude of those peaks decreases as the temperature increases, see Figure 15. Both plots, which display a change of convexity at some temperature, were reasonably well fitted using Boltzmann function (Origin 9.5):

(9) $$f\left(T\right)=A_{2}+\frac{A_{1}-A_{2}}{1+\exp\left(\frac{T-T_{0}}{dT}\right)},$$

where $A_{1},A_{2},dT,T_{0}$ denote the parameters of the fit. The points of inflection are the solutions of the equation:

(10) $$\frac{d^{2}}{dT^{2}}f\left(T\right)=0,$$

and read $T_{0}=0.23$ for $\bar{V\left(\vec{r}\right)}$ and $T_{0}=0.22$ for $\bar{D\left(\vec{r}\right)}$. Those temperatures are interpreted as the estimations of $T_{g}$. They are, again, in a good agreement with previous MC-based estimations of $T_{g}$.

## 4. Discussion

The study was oriented onto the characterization of local Monte Carlo dynamics/kinetics of an equilibrium BFM polymer matrix in MC-time interval $\left(5-25\right)\cdot 10^{4}$ MCS, typical for MC simulations of various non-linear optical phenomena [69]. It constitutes a follow-up of the static characterization recently reported in Refs. [69,71].

The polymer dynamics at point $\vec{r}$ and time *t* was characterized at three temperatures (below, above and close to the glass transition), using MC-time dependent parameters $V(\vec{r},t)$, $C(\vec{r},t)$ and $D(\vec{r},t)$ corresponding, respectively, to local free volume, local mobility, and their product. Special emphasis was put on the dynamics of local volume $V(\vec{r},t)$, because it plays the main role in the kinetic model used in MC simulations [69]. To this end, basic probabilistic concepts were used, including probability distributions of various instantaneous and time-averaged parameters. Additional information, of a rather qualitative character, followed from 2D instantaneous maps and short time series.

An extensive discussion of the results was already presented in the main text. In what follows, we discuss them in a wider context.

The paper offers a semi-quantitative support to the physical picture of a spatio-temporal heterogeneity of the polymer (BFM) matrix. The spatial heterogeneity is characterized by instantaneous maps of local free volume (Figure 1), local mobility (Figure 7) and their product (Figure 11), as well as by their probability distributions (Figure 8 and Figure 12). The temporal heterogeneities are illustrated by a highly irregular short time series (Figure 3 and Figure 10).

The main result of the study is the estimation of both *maximal* and *typical* time scales characteristic of various structural processes. The general conclusion is that the *typical* time scales are of an order of magnitude $10^{3}$ MCS, up or down, around two orders of magnitude smaller than the period of the simulations of non-linear optical phenomena. Namely, at a high temperature (T=0.5) the time of life of a vast majority (99%) of the V=6 and V=0 cells does not exceed a few hundred MCS. In the glass transition region, it becomes approximately three times larger. Similar increase, ascribed to the slowing-down phenomenon, was found in the simulations of an all-optical poling effect [79]. Another approach, based on Central Limit Theorem, provides a rough estimation of the time of life of an arbitrary value of parameters $V(\vec{r})$ or $C(\vec{r})$ at cell $\vec{r}$. The corresponding time scales read a few thousand MC steps. At a low temperature (T=0.1) the local dynamics is substantially frozen. 60% of the cells preserve the values of *V* during the simulation period, among them 78% of the population of blocked cells (V=0). Close to the glass transition the concentration of frozen cells drops down to a few percent. The *maximal* time-scales correspond to a negligible concentration of the cells, and are substantially larger. The times of life of V=6 and V=0 cells constitute, away from the glass transition region, a few thousand and a few ten of thousand MCS, respectively. Close to the glass transition they increase, at least by one order of magnitude, and become comparable to the full period of the simulations.

Another issue of interest is the joint effect of a small amount of local free volume and of a low local mobility at point $\vec{r}$, characterized by parameter $D(\vec{r},t)$, which promotes or hinders orientational diffusion of guest molecules in MC modeling. We have found that orientational diffusion is fully blocked (D=0) for 75−80% of the cells at temperatures up to $T_{g}$; at a high temperature half of the cells do not support any orientational diffusion.

The fields $V(\vec{r},t)$ and $D(\vec{r},t)$ offer an elegant geometric criterion (inflection point in Figure 15) for the localization of $T_{g}$, based on those parts of the probability distributions which characterize the absence of rotational diffusion in MC modeling.

It is generally accepted that a polymer matrix is, to some extent, frozen at low temperatures. This study offers a simplified, but nevertheless quantitative physical picture of the dynamics of guest molecules dispersed in the BFM matrix at low temperature (T=0.1). It is based on the observation that approximately 60% of the cells preserve their local volume *V* during the simulation (cases V=0,6 were discussed in Section 3.1). Thus, around 40% of the cells change local volume *V*, among them fully blocked cells (V=0) which, as a rule, after a short time return to fully blocked state. They constitute approximately 15% of all the cells and, correspondingly, around 75% of the cells support local dynamics in a fixed local environment in the course of the simulation. Moreover, since around 80% of the cells do not support any orientational diffusion, its impact on an overall dynamics becomes rather limited. All this leads to the *quasi-frozen configuration* physical picture in which an orientational diffusion is neglected and the dynamics takes place in time-independent local environment $V(\vec{r})$, set by an initial configuration. The active dynamics can be tentatively ascribed to large clusters of cells with V=6. This physical picture offers a plausible starting point for a stochastic modeling of the dynamics of guest molecules at low temperatures.

This study yields some preliminary contributions to the physical picture of the dynamical aspects of *quasi-binary mosaic states* studied previously in the static regime [69,71]. The next steps toward the characterization of random fields (stochastic processes) $V(\vec{r},t)$, $C(\vec{r},t)$ and $D(\vec{r},t)$ require addressing two main issues. We are of an opinion that the most important one is the study of the cross-correlations of those fields in space and time. The former includes the study of the correlations between times of life of a cell and a large cluster membership, in the context of a hypothetical but plausible clusterization of long-lived areas. The temporal correlations concern, first of all, those at fixed point $\vec{r}$. This approach was successfully used in the analysis of structural correlations in two-dimensional model liquids [72]. The second issue is the concept of *typical* time scales, which should be analyzed in more detail, using advanced mathematical methods for time-series analysis. It includes, in particular, the characterization of times of life of the clusters of cells, especially those with V=6. Some of those topics are at progress now.

Finally, let us point out that the time scales, in particular the probability distributions of relaxation times, play one of the central roles in modeling of the complexity which accompany relaxation processes [74]. This study has introduced some parameters and methods which can be useful for a validation of model probabilistic concepts [74] in physical systems. In particular, we have found some traces of temporal heterogeneity in BFM matrix.

This study does not focus on any specific type of polymer system. However, the model might be expanded onto various materials and problems [93,94]. 

## Data Availability

The data presented in this study are available on request from the corresponding author.
